# Unraveling the Complex Genomic Interplay of Sickle Cell Disease Among the Saudi Population: A Case-Control GWAS Analysis

**DOI:** 10.3390/ijms26062817

**Published:** 2025-03-20

**Authors:** Ali Alghubayshi, Dayanjan Wijesinghe, Deemah Alwadaani, Farjah H. Algahtani, Salah Abohelaika, Mohsen Alzahrani, Hussain H. Al Saeed, Abdullah Al Zayed, Suad Alshammari, Yaseen Alhendi, Barrak Alsomaie, Abdulmonem Alsaleh, Mohammad A. Alshabeeb

**Affiliations:** 1 Department of Clinical Pharmacy, College of Pharmacy, University of Ha’il, Ha’il 55473, Saudi Arabia; a.alghubayshi@uoh.edu.sa; 2 Department of Pharmacotherapy and Outcomes Science, School of Pharmacy, Virginia Commonwealth University, Richmond, VA 23298, USA; wijesingheds@vcu.edu (D.W.); souad.hamed@nbu.edu.sa (S.A.); 3 Medical Genomics Research Department, King Abdullah International Medical Research Center (KAIMRC), Riyadh 11481, Saudi Arabia; alwadaanide@mngha.med.sa; 4 King Saud bin Abdulaziz University for Health Sciences (KSAU-HS), Ministry of National Guard Health Affairs (MNGHA), Riyadh 11426, Saudi Arabia; alzahranimo5@mngha.med.sa (M.A.); alhendiy@kaimrc.edu.sa (Y.A.); somaieb@mngha.med.sa (B.A.); alsalehabd@kaimrc.edu.sa (A.A.); 5 Hematology/Oncology Center, King Saud University Medical City (KSUMC), Riyadh 11411, Saudi Arabia; falgahtani@ksu.edu.sa; 6 Research Department, Qatif Central Hospital (QCH), Qatif 32654, Saudi Arabia; sabohelaika@moh.gov.sa; 7 Pharmacy Department, Qatif Central Hospital (QCH), Qatif 32654, Saudi Arabia; 8 King Fahad Hospital, Ministry of National Guard Health Affairs (MNGHA), Riyadh 11426, Saudi Arabia; 9 Hematology Department, Qatif Central Hospital (QCH), Qatif 32654, Saudi Arabia; alsaeedhha@hotmail.com (H.H.A.S.); abalzayed@moh.gov.sa (A.A.Z.); 10 Department of Clinical Pharmacy, College of Pharmacy, Northern Border University, Rafha 91911, Saudi Arabia; 11 Saudi Biobank Center, King Abdullah International Medical Research Center (KAIMRC), Riyadh 11481, Saudi Arabia; 12 Operations Department, King Abdullah International Medical Research Center (KAIMRC), Riyadh 11481, Saudi Arabia; 13 Blood and Cancer Research Department, King Abdullah International Medical Research Center (KAIMRC), Riyadh 11481, Saudi Arabia; 14 Pharmaceutical Analysis Department, King Abdullah International Medical Research Center (KAIMRC), Riyadh 11481, Saudi Arabia

**Keywords:** sickle cell disease (SCD), olfactory receptor clusters (ORs), genome-wide association study (GWAS), Saudis, β-globin subunit, hemoglobin S (HbS)

## Abstract

Sickle cell disease (SCD) is a severe inherited blood disorder characterized by abnormal hemoglobin (HbS) that leads to varying degrees of severity, including chronic hemolysis, episodic vaso-occlusion, and damage to multiple organs, causing significant morbidity and mortality. While SCD is a monogenic disease, its complications are influenced by polygenic factors. SCD prevalence is notably high in regions including the Middle East, with Saudi Arabia reporting significant cases, particularly in the Eastern Province. Most genetic factors associated with SCD outcomes have been identified in populations predominantly from Africa or of African ancestry. This study aims to identify genetic variants that characterize Saudi SCD patients with the potential to influence disease outcomes in this population. A multicenter case-control genome-wide association study (GWAS) was conducted involving 350 adult Saudi SCD patients and 202 healthy controls. Participants were genotyped using the Affymetrix Axiom array, covering 683,030 markers. Rigorous quality control measures were applied to ensure data integrity. Fisher’s exact was used to identify genetic variants with a significant difference in allele frequency (*p* < 5 × 10^−8^). Functional annotations and regulatory functions of variants were determined using the Ensembl Variant Effect Predictor (VEP) and RegulomeDB databases. The GWAS identified numerous significant genetic variants characterizing SCD cases in the Saudi population. These variants, distributed across multiple chromosomes, were found in genes with known functional consequences. A substantial proportion of the markers were detected in the olfactory receptor cluster, *TRIM* family, and *HBB* locus genes. Many of the identified genes were reported in previous studies showing significant associations with various SCD outcomes, including hemoglobin regulation, inflammation, immune response, and vascular function. The findings highlight the genetic complexity underlying SCD and its clinical manifestations. The identified variants suggest potential molecular biomarkers and therapeutic targets, enhancing our understanding of the molecular basis of SCD in the Saudi population. This is the first genetic analysis characterizing SCD patients compared to healthy individuals, uncovering genetic markers that could serve as diagnostic biomarkers and therapeutic targets. Given the known molecular mechanisms of the detected genetic loci, these provide a foundation for precision medicine in SCD management, highlighting the need for further studies to validate these results and explore their clinical implications.

## 1. Introduction

Sickle cell disease (SCD) is an inherited red blood cell disorder in which the abnormal sickle hemoglobin leads to shortened red blood cell survival. SCD is a major global public health concern, characterized by a significant rise in prevalence worldwide. It has demonstrated a 41.4% increase from 2000 to 2021. Over this period, the number of SCD cases grew from 5.46 million to 7.74 million, causing approximately 376,000 annual deaths due to associated complications [1]. By 2050, it is predicted that the number of individuals with SCD will surge by 30% compared to current numbers [2]. SCD is prevalent among populations from Africa, India, the Caribbean, the Middle East, and the Mediterranean regions. Among the Middle Eastern Arab countries, SCD prevalence varies, with particular nations exhibiting amplified rates [3,4]. In the Gulf, specifically Saudi Arabia, SCD occurrence ranges from 2.6% to 4.2%, affecting 1 in 80 people in certain areas, with a 0.73% mortality rate owing to various complications [5,6,7]. However, a significant proportion of Saudis, notably those in the Eastern province (24%), either have the disease or carry one copy of the genetic mutation [8,9].

SCD arises from a non-synonymous missense single nucleotide polymorphism (SNP) [*rs334* c.20 A>T] in the *HBB* gene encoding the beta-globin protein, representing the fundamental basis underlying SCD pathology. This SNP substitution (adenine to thymine) in the sixth codon of *HBB* replaces the glutamine amino acid (encoded by GAG) with valine (encoded by GTG) in the translated protein. This missense mutation precipitates abnormal hemoglobin polymerization under low oxygen conditions, ultimately engendering the signature sickle morphology of red blood cells and manifesting the downstream clinical phenotype [10]. Sickling impedes oxygen transport and predisposes cells to hemolysis. Additionally, vascular obstructions and circulation of irregular cell adhesion molecules decrease vascular endothelial cell function, increase blood coagulation elements, and amplify inflammation biomarkers. These factors underlie classic complications such as painful ischemic crises, strokes, acute chest syndrome, and multiorgan damage, characterizing the spectrum of disease severity [11]. Despite SCD being a recognized Mendelian genetic disorder exhibiting an autosomal recessive inheritance pattern, it demonstrates complex phenotypes shaped by multiple genes [10,12,13].

Despite the acknowledged necessity to characterize genetic variants linked to SCD outcomes across populations, the current literature remains deficient [14,15,16,17,18,19,20,21]. These variants, potentially modifying SCD severity, are integral for elucidating phenotypic diversity. As observed in hemoglobin-associated or subsidiary hemoglobin regulatory genes, they may exacerbate or mitigate clinical manifestations. Therefore, identifying mutations is imperative for detecting clinically significant markers and elucidating the biological pathways potentially linked to the varying burden and severity of SCD manifestations, stratifying patient risk and offering new therapeutic targets [5,22,23].

Earlier SCD genetics research predominantly utilized candidate gene approaches, focusing on particular populations, especially those of African ancestry [12,13,24]. This highlights a significant gap in the literature and underscores the need to identify more genetic signals potentially linked to SCD outcomes across diverse populations. Such knowledge is crucial for implementing personalized therapy for SCD patients and offers insights into the role of these variants in the disease’s pathology [25]. The limited understanding of the genetic underpinnings of SCD, especially in underrepresented groups such as the Saudi population, presents a primary barrier to managing SCD complications in these individuals. This underscores the importance of identifying additional genetic markers that may influence SCD outcomes. Conducting genome-wide association studies (GWASs) is a broader genetic approach that could reveal additional insightful markers among this population compared to previously used candidate gene analyses.

Interestingly, in a previous GWAS, our research group identified several markers influencing thromboembolic risk in Saudi SCD patients, distinct from those previously identified in other populations [26]. This suggests that Saudi SCD patients may carry unique genetic markers that potentially explain the severity of complications. To our knowledge, no studies have yet compared SCD patients with healthy individuals using GWAS to identify distinctive genetic differences between cases and controls. This project aims to (i) explore the genetic makeup of the Saudi population with SCD and (ii) utilizing the literature, identify the biological pathways and molecular mechanisms associated with disease manifestations. The knowledge gained is expected to construct personalized prognostic models, identify critical targets for future drug development, and provide information on stratified therapeutic strategies tailored to Saudi patients’ genetic makeup.

## 2. Results

### 2.1. Study Participant Characteristics

**Figure 1:**
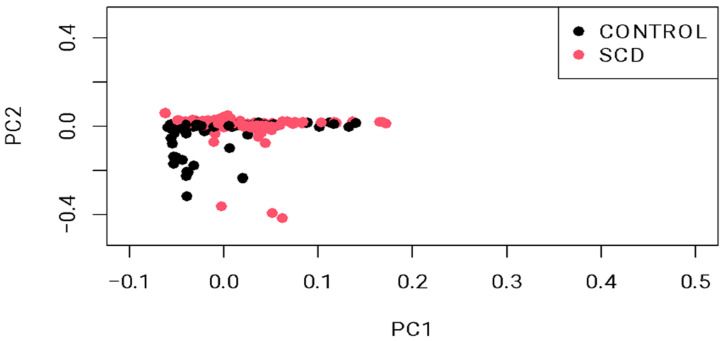
**Principal component analysis (PCA) plot showing sample stratification.** The permutation test (T1) confirmed the sample heterogeneity (*p* = 0.988).

**Figure 2:**
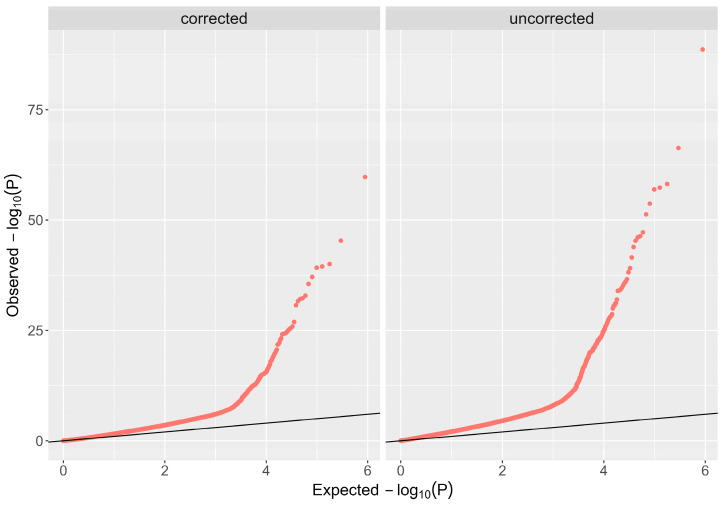
**Corrected Q-Q plot.** The Bayesian method (based on the BACON algorithm) was used to control inflation; the lambda (λ) value was reduced from 3.0 to 1.2.

The demographic characteristics of the 552 enrolled subjects are summarized in Table 1. Age matching between cases and controls revealed mean values of 32.7 ± 10.2 years for cases versus 29.4 ± 8.4 years for controls. For gender, both cases and controls exhibited a male distribution of 54% and 51%, respectively. Principal component analysis revealed distinct clusters representing genetic variation between cases and controls (Figure 1). Scatter plot visualization of genotype data showed homogeneous dispersion for both groups, further validated by a permutation test (T1) yielding *p* = 0.988, which negates potential population stratification. Additionally, the genomic inflation rate was corrected using BACON, represented by a decreased lambda (λ) from 3.0 to 1.2 (Figure 2).

### 2.2. Top Identified Variants with Functional Consequences

**Figure 3:**
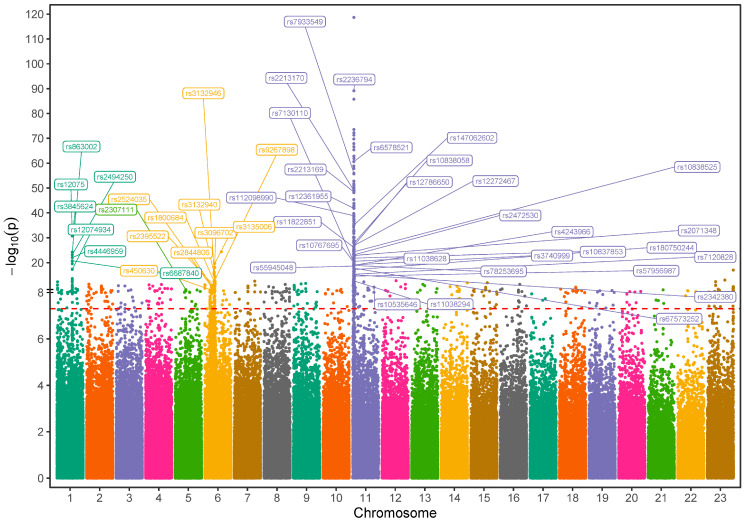
**Manhattan plot visualization of genome-wide association study (GWAS) results in Saudi SCD cohort versus healthy Saudi individuals**: The SNPs that met the genome-wide significance threshold (*p* < 5 × 10^−8^) are shown just above the red-dotted line. The SNPs were plotted in different colors to show a distinction between the chromosomes.

This GWAS analysis identified 48 variants with functional significance (*p* < 5 × 10^−8^) located within or nearby 31 known distinct genes. These variants, comprising both coding and regulatory non-coding markers, persisted after rigorous quality control measures and spanned over multiple chromosomes (Figure 3, Table 2). Notably, gene-based mapping revealed that approximately 23% of implicated variants clustered within olfactory receptor gene regions on chromosome 11, which are known to regulate hemoglobin β expression (Supplementary Table S1). Specifically, we pinpointed high allele frequencies in coding mutations, including two missense variants: rs7933549 (MAF = 0.48) in *OR51V1*, rs2472530 (MAF = 0.45) in *OR52A5*, and a frameshift variant rs147062602 (MAF = 0.35) in *OR51B5*. In contrast, low inheritance levels were observed in a missense variant, rs12361955 (MAF = 0.077) in OR51S1, and a frameshift variant, rs112098990 (MAF = 0.029) in *OR52A1* with a statistically significant difference. Numerous other hemoglobinopathy-associated loci have emerged, such as rs2213169, rs2213170, and rs7130110 markers, which are neighbors to *HBE1* and *HBG2* genes. The frequencies of these alleles were found to be 43.7–46.6% in the SCD cohort compared to 1.5–1.8% in the healthy cohort. Additionally, a few common variants in SCD cases were mapped to tripartite motif (*TRIM*) gene family members on chromosome 11, such as *TRIM5*, with two missense mutations rs10838525 and rs11038628 (MAF = 0.27 vs. 0.05 and 0.33 vs. 0.07, respectively). Beyond these, several other markers within diverse genes across chromosome 11, including *RRM1*, *STIM1*, and *MMP26* (Table 2), are more common among SCD individuals.

Outside chromosome 11, candidate variants showed genome-wide significance as well, including missense variants rs12075 in *ACKR1* on chromosome 1, rs2307111 in *POC5* on chromosome 5, and rs450630 in *SCAND3* on chromosome 6. Furthermore, various regulatory variants located on chromosome 6 within the *HLA* complex, *NOTCH*, and *AGER* genes were also identified. The phenotypes associated with the identified genes are described in Supplementary Table S1, as reported in the literature. This table summarizes the potential pathways linked with hemoglobinopathies for each detected gene or gene family.

## 3. Discussion

The application of GWAS has revolutionized our understanding of complex diseases by identifying genetic variants associated with disease risk across the genome [27]. Despite extensive efforts, the current literature on genetic factors beyond the known HBB loci that explain SCD outcomes remains insufficient [12]. Identification of these genetic variants is crucial for understanding the molecular basis of SCD and its phenotypic diversity and is essential for the implementation of personalized therapy. Previous GWASs have identified several associations between a few genetic markers and different SCD phenotypes, such as *rs3115229*, linked to vaso-occlusive pain episodes, and markers (e.g., rs766432 and rs1427407) near *BCL11A* and rs9494145 in *HBS1L-MYB*, correlated with variable fetal hemoglobin (HbF) expression [14,28]. Moreover, a GWAS analysis conducted on Saudi SCD patients identified seven markers associated with thromboembolic events, highlighting a significant risk in this population [26]. Building on these findings, the GWAS data were further analyzed to identify additional genetic variants that distinguish SCD patients.

This study pinpointed several noteworthy markers, and 62.5% of them are located on chromosome 11, with most of them (70%) mapped to the olfactory receptor (*OR*) gene cluster, *TRIM* family, and *HBB* locus genes. The *OR* genes, which constitute a significant portion of the mammalian genomes, are pseudogenes—a non-functional DNA segment [29]. Pseudogenes have no direct contribution to phenotypic traits; however, they tend to regulate the expression and function of genes. Numerous mutations in these genes may potentially affect phenotypic diversity through various mechanisms, such as impacting mRNA translation and manipulating epigenetic remodeling [26,30]. Notably, the *OR* gene family is aberrantly expressed in erythroid cells and located near the β-globin gene cluster. This proximity suggests a regulatory role in hemoglobinopathies, as these genes may modulate the chromatin structure at the CTCF binding site within the β-globin gene. This genetic linkage has been confirmed in both clinical and laboratory studies [31,32,33,34]. Also, various studies connect *OR* polymorphisms to SCD morbidity [12]. In our study, we observed a high prevalence of two missense variants: rs2472530 in *OR52A5* and rs7933549 in *OR51V1*, and a frameshift variant rs147062602 in *OR51B5* gene in SCD patients compared to the controls (MAF = 0.45, 0.48, and 0.35 among SCD patients versus 0.15, 0.015, and 0.018 in the controls, respectively). These variants have been previously reported as risk markers for thromboembolic events in Saudi SCD patients [26]. Additionally, we identified a novel frameshift variant, rs112098990, in the *OR52A1* gene, which typically introduces a stop codon, terminating the DNA sequence. Previously, an upstream variant rs4910715 in *OR52A1* was identified as part of a haplotype that characterizes classical sickle beta-globin [35]. *OR51V1* was also reported in a previous study linked to low HbA2 levels in healthy adults [36]. Another coding variant, rs12361955 in the *OR51S1* gene, was detected less frequently in our SCD cohort suggesting a protective effect. These findings are consistent with previous reports linking different mutations in OR cluster genes with multiple SCD-related phenotypes. These include hemolysis, variability in HbF levels, inflammation, hemostasis, and various hematological traits [12]. The reported data suggests a role for OR genes in regulating β-globin gene clusters that possibly influence SCD phenotypes.

Our findings showed significant allele differences in nine variants in four *TRIM* family genes (*TRIM5*, *TRIM6*, *TRIM22*, and *TRIM34*), and two of them are missense variants: rs10838525 and rs11038628, located in *TRIM5*. The TRIM protein family is involved in immune modulation, cell-cycle progression, inflammation, oxidative stress, and hematopoietic stem-cell differentiation. All these mechanisms are critical for SCD complications. TRIM proteins play an important role in modulating TGF-β-activated kinase 1 (TAK1) to induce NF-κB and MAP kinase signaling. Kinase activation promotes NF-κB activation through IKKβ ubiquitination, regulates AP-1 signaling, and induces antiviral responses and innate immune activities [37]. *TRIM5*, for instance, which encodes immunomodulatory proteins, plays a defensive role against retroviral infections, while *TRIM6* is crucial in regulating stem-cell proliferation. Although neither *TRIM5* nor *TRIM6* are directly linked to hemoglobin expression—unlike *TRIM28*, which affects erythrocyte development as shown in animal models—their immune modulatory roles suggest they have an indirect influence on hematological outcomes. Thus, TRIM proteins were implicated in SCD pathology [12]. Furthermore, the previously reported GWAS data showed numerous associations between several loci in *TRIM* genes and a list of hematological and inflammatory traits. Notably, rs17305868 and rs11601507 in *TRIM5* indicated strong associations with increased platelet counts (*p* = 9 × 10^−10^) [38] and coronary artery disease (*p* = 6 × 10^−13^) [39], respectively. These pathophysiological phenotypes are common in SCD.

Other functional markers, with high prevalence in the SCD cohort, were detected across several genes throughout the β-globin locus on chromosome 11, such as rs2071348, rs2213169, rs2213170, and rs7130110. These variants have previously been linked to various hematological traits, such as hemostasis and thromboembolism, in SCD patients [26,40,41]. For example, rs7130110 in *HBE1* was identified as a marker impacting HbF levels and predicting the efficacy of hydroxyurea treatment in managing SCD [42,43]. Another variant (rs2213169) in *HBE1* was previously reported as a factor associated with reduced hematocrit volume [41]. These findings provide strong evidence suggesting *HBE1* as a potential pharmacogenomic marker for the treatment of SCD [43]. Moreover, two regulatory variants (rs6578521 and rs1182285) were identified in the *MMP26* gene, which encodes matrix metallopeptidase 26. This gene plays a crucial role in the degradation of extracellular matrix (ECM) components, an important process in angiogenesis, inflammation, and tissue remodeling [44]. Previous studies have shown that certain mutations in this gene are linked to hematological and neurological conditions, explained by ischemic stroke events [12]. Other related conditions, such as pneumonia risk and variability in cognitive abilities, have also been reported in the GWAS catalog database [45,46] (Supplementary Table S1). These traits are frequent too among SCD patients. Some interesting functional variants (rs55945048 and rs10535646), mapped to the *RRM1* and *SIDT2* genes, respectively, were also identified in our study. The dysregulation of these genes might have a direct or indirect impact on various SCD outcomes. For instance, RRM1 encodes ribonucleotide reductase, a rate-limiting enzyme in DNA synthesis, whereas hydroxyurea is a specific inhibitor of this enzyme [47]. Thus, significant links were found between the *RRM1* gene overexpression and hydroxyurea resistance [48,49], potentially due to increased enzyme activity overcoming hydroxyurea’s inhibitory effects. A previous study reported that 18% of SCD patients discontinued taking this medication for various reasons, not explicitly indicating resistance. These reasons include the drug’s immunosuppressive effects, failure to improve HbF levels, and likely also non-compliance [50,51]. Genetic factors, such as the carriage of certain *RRM1* mutations, may partially explain the variability in responses of patients to hydroxyurea. Another important marker characterizing SCD patients is rs10767695 in the *STIM1* gene, with a double prevalence of the variant allele among the SCD group (60%) compared to the healthy cohort (30%). A previous study linked this variant to mean platelet volume [52]. The markers in *MMP26, RRM1, SIDT2*, and *STIM1* align with assorted molecular pathways linked to endothelial dysfunction, inflammation, vascular disease, and numerous SCD-associated complications [44,48,49,53,54] as described in Supplementary Table S1.

Beyond chromosome 11, three coding variants (missense) were found to be more common among the SCD cohort involving rs12075 in *ACKR1* on chromosome 1, rs2307111 in *POC5* on chromosome 5, and rs450630 in *SCAND3* on chromosome 6. The dysregulation of these genes was associated with various SCD outcomes [55,56,57,58]. The *ACKR1* gene, also known as the Duffy Antigen Receptor for Chemokines (DARC), is expressed on red blood cells and endothelial cells and plays an important role in modulating inflammation and chemokine levels. It may specifically modulate leukocyte trafficking and is predicted to be involved in various homeostasis mechanisms underlying a variety of SCD phenotypes. For example, it has been reported to potentially influence the severity of organ damage associated with SCD, including anemia, leg ulcers, priapism, and kidney dysfunction [12,55]. In addition, previous studies have demonstrated that numerous genetic variations in *ACKR1*, including the detected missense mutation rs12075 (G>A) in our study, are significantly associated with the inflammatory cascade among European populations [59]. The wild type allele (G) predisposes patients to a severe pattern of SCD. Our data revealed that this allele is more prevalent among the SCD cases. A previous study suggested a role for rs3845624 in regulating C-reactive protein levels in plasma and rs2494250 in (C-C motif ligand 2) CCL2 measurement [59,60]. CCL2 is a chemotactic factor, known as monocytic chemotactic protein 1 (*MCP-1*), which employs macrophages to induce the immune response. CCL2 and its receptors (ACKR1 and ACKR2) impact disease outcomes complementarily [61]. Additionally, the missense variant rs450630 in *SCAND3* was reported recently as a marker associated with decreased hemoglobin concentration (*p* = 6 × 10^−11^) [58].

Several regulatory variants that possibly contribute to variable disease phenotypes were detected in our study. These include the intergenic variant rs3845624 located between the *MPTX1* and *CADM3*-AS1 genes, linked with inflammation and adhesion regulations, and the intronic SNP rs2494250 located near *FCER1A* gene. As the *FCER1A* gene encodes the alpha chain of the high-affinity IgE receptor (FcεRI), it plays a role in allergic responses. This explains the reported association between *rs2494250* and the non-immediate urticaria/angioedema (NIUA), a type of delayed hypersensitivity reaction (DHR) that can occur hours to days after exposure to a triggering substance, particularly nonsteroidal anti-inflammatory drugs (NSAIDs) [62,63]. DHR can be life-threatening, particularly in the context of transfusion complications in SCD patients, and typically occurs 5–10 days following transfusion. These reactions often result in decreased hemoglobin levels and pain crises [64]. The incidence of DHR related to blood transfusion (alloimmunization) seems to be notably high (16.7%) in Saudi SCD patients [65]. Other identified variants with high regulatory function scores were notable across chromosome 6. Most of these markers were mapped to the *HLA* gene family, *AGER*, and *NOTCH* genes. The ontological pathways connecting these genes to SCD complications can be explained through their roles in regulating inflammation, immune responses, and endothelial function. A linkage was detected previously between several markers in these genes with various SCD phenotypes, such as vaso-occlusive crisis and organ damage [12]. Among these markers, the SNP *rs3135006* located in the *HLA-DQB1* gene was reported earlier as a factor for increased ratio levels of cell adhesion proteins [Activated Leukocyte Cell Adhesion Molecule (ALCAM)/Cadherin 5 (CDH5)] [66]. In addition, the detected variant rs2524035 in *HLA-G* was demonstrated previously as a factor associated with decreased lymphocyte levels (*p* = 3 × 10^−23^) [67]. Moreover, previous reports highlighted several *HLA* variants that are linked with the risk of stroke, acute chest syndrome (ACS), pain episodes, cholelithiasis, and hyperbilirubinemia in SCD patients [12,13].

Another notable marker in our study (rs1800684) in the *AGER* gene was nominated in a previous GWAS study as a factor associated with a higher expression of the Transforming Growth Factor Beta Receptor 2 (TGFBR2) (*p* = 3 × 10^−13^) [66]. Genetic mutations in both AGER and TGF-β receptors have been previously shown to increase the risk of various SCD complications, including stroke, priapism, infection, avascular necrosis, pulmonary hypertension, acute chest syndrome, and acute pain crises [12,13]. Additionally, four variants in *NOTCH4* on chromosome 6 were detected. This gene plays an important role in regulating endothelial function, inflammation, and hematopoiesis, protecting against endothelial dysfunction and apoptosis and balancing proinflammatory and inflammatory responses through various signaling pathways, with notable anti-inflammatory effects by reducing proinflammatory cytokine expression and co-stimulatory protein levels [68,69]. Among the identified variants, *rs3132940* was found to be associated with an increased risk of sarcoidosis disorder, specifically non-Lofgren’s syndrome without extrapulmonary manifestations [70]. This serious condition was reported previously in SCD patients, noting that patients suffering from ACS and sarcoidosis had a higher mortality rate [71].

This study represents the first GWAS analysis comparing SCD patients with healthy individuals, with a particular focus on the Saudi population. It included the largest genome scan of Saudi SCD patients to date. The study identified SCD distinguishing markers, suggesting their potential as powerful diagnostic biomarkers and therapeutic targets. The identified genetic variants may influence SCD pathophysiology through potential alterations in gene regulation, including changes in transcription factor binding, gene expression levels, or mRNA splicing patterns. These molecular changes could affect vascular function, inflammatory responses, and related physiological processes relevant to SCD. The co-occurrence of potentially functional coding and non-coding variation across these priority candidate genes emphasizes their likely role in modulating SCD phenotypic expression through diverse molecular genetic mechanisms. Collectively, these data implicate a complex interplay among variants distributed across the genome and concentrated in key genes that may contribute to driving SCD pathophysiology through a multitude of effects.

## 4. Materials and Methods

### 4.1. Sample Recruitment

**Figure 4:**
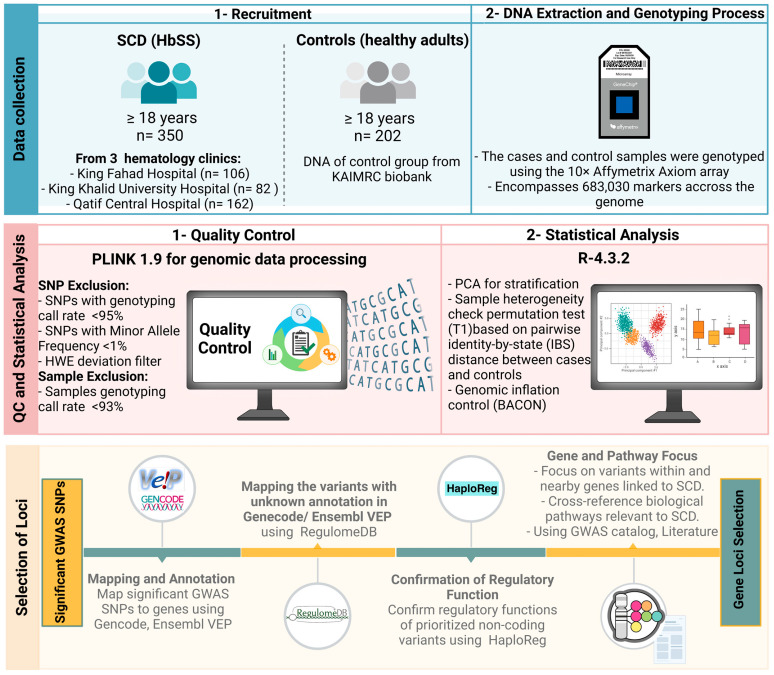
Integrated workflow for enrollment, DNA extraction, quality control, and statistical analysis in a GWAS of Saudi sickle cell disease.

As summarized in Figure 4, this multicenter case-control study enrolled 350 unrelated adult SCD subjects (cases) and 202 healthy adults (controls), aged ≥18 years, encompassing both males and females. SCD diagnosis was confirmed via positive sickling assay and verification of homozygosity for the causative *rs334* A>T mutation. Participating patients routinely attended one of three hematology clinics: King Fahad Hospital (KFH) in Riyadh under the Ministry of National Guard Health Affairs (MNGHA), contributing 106 cases; King Khalid University Hospital (KKUH) in Riyadh under King Saud University Medical Center (KSUMC), contributing 82 cases; and Qatif Central Hospital (QCH) in the Eastern Province under the Ministry of Health, contributing 162 cases. The 202 control specimens were obtained from the KAIMRC biorepository. The study predominantly focused on HbSS homozygotes, excluding other SCD genotypes like HbSC, HbS-beta-thalassemia, HbSD, and HbSO, which were identified through hemoglobin electrophoresis. Notably, a majority of KKUH and KFH Riyadh patients were referrals from the Saudi Arabian southwestern or northern regions.

### 4.2. Genomic Analysis and Quality Control (QC)

The cases and control samples were genotyped using the Affymetrix Axiom array (Axiom 2.0 reagent kit designed by Applied Biosystems^TM^, Waltham, MA, USA, catalog number #901758), which encompasses 683,030 markers for the GWAS. All details regarding the genomic analysis, which includes sample processing, genotyping, quality control call rates (CRs), validity, and reliability of the platform used, have been previously published [26]. Several quality control tests were applied to filter out low-quality genotyping data. SNPs that had a genotyping call rate < 95%, a minor allele frequency (MAF) < 1%, or a Hardy–Weinberg equilibrium (HWE) *p*-value < 0.05 were excluded. In addition, samples with genotyping CR < 93% were omitted.

### 4.3. Statistical Analysis

Plink software version 1.9 was employed for genomic data processing. Genotype differences between groups were estimated using a Fisher’s exact test. For GWAS, a threshold of *p* < 5 × 10^−8^ was established to identify loci with a statistically significant difference. Mean and standard deviation calculations were performed to evaluate age matching. R statistical package (qqman) version R-4.3.2. was used to generate Manhattan, quantile-quantile (Q-Q), and principal component analysis (PCA) plots. Population stratification was also assessed, with sample heterogeneity evaluated through the permutation test (T1) based on pairwise identity-by-state (IBS) distance between cases and controls. Additionally, the Bayesian method, utilizing the BACON (Bayesian Analysis Computation and Optimization) standard, was used to control the genomic inflation rate.

### 4.4. Selection of Loci

The identified signals were mapped to genes based on their original annotations and the Gencode [72]. The Ensembl Variant Effect Predictor (VEP) tool was then used to confirm annotation and prioritize the identified markers based on their predicted effects [73]. The VEP is a robust and well-established tool that uses automated annotation pipelines based on experimental evidence highlighting consequence types and gene biotypes. The VEP tool was also used to determine the functionality of variants located in non-coding regions. We further utilized the RegulomeDB database to annotate variants that were not detected by VEP. RegulomeDB enabled us to prioritize the identified variants based on their potential regulatory function; it provides ranks for the screened variants [74]. Variants with high ranks, indicating strong evidence of functionality, were selected. Furthermore, the HaploReg database was used as a confirmatory step to identify the regulatory functions of non-coding variants [75]. The variants located within or near known genes and potentially linked to a pathological mechanism or outcomes of SCD were selected. Due to the lack of access to phenotypic data in our SCD cohort, we cross-referenced biological pathways linked to candidate genes using the literature and previously identified phenotypes indicated in the GWAS catalog website [76]. We hypothesized their relevance to SCD outcomes based on known molecular functions and ontologies curated in databases related to blood disorders and SCD phenotypes. Furthermore, genes identified with numerous associated SNPs, including both coding and non-coding variants with functional consequences, were selected as high priority markers.

## 5. Conclusions

This GWAS has uncovered significant genetic markers in Saudi SCD patients across multiple chromosomes. Our findings provide novel insights into SCD genetics in the Saudi population, particularly involving key gene families dominant in the disease cohort. A substantial portion of our findings aligns with genes previously implicated in various SCD complications, lending support to the validity of our approach. These results contribute to the broader understanding of SCD’s molecular basis and underscore the importance of population-specific genetic factors in SCD research. The identified markers hold potential for informing future personalized medicine approaches in SCD management. Replication studies in independent cohorts are essential to validate these results and exclude potential technical or methodological biases. Furthermore, functional studies are needed to elucidate the mechanisms underlying disease severity and complications. This work represents a significant step toward comprehending the genetic landscape of SCD in the Saudi population and may guide future diagnostic and therapeutic strategies.

### Limitations

While this study provides valuable insights into the genetic landscape of SCD in the Saudi population, several limitations should be noted. The sample size, although substantial, may not capture all possible genetic variations in the Saudi SCD population. The lack of detailed phenotypic data limits our ability to directly link genetic variants to specific SCD complications or severity levels. Additionally, the focus on the Saudi population may limit the generalizability of our findings to other ethnic groups. Furthermore, while we identified potentially functional variants, experimental validation of their biological effects was beyond the scope of this study. Our GWAS approach may not capture all relevant genetic variations, particularly rare variants.

## Figures and Tables

**Table 1:** Study participant characteristics.

Characteristic	Cases (n = 350)	Controls (n = 202)
Age (mean ± SD)	32.7 ± 10.2	29.4 ± 8.4
Male/female	195 (56%)/155 (44%)	103 (51%)/99 (49%)

SD = standard deviation.

**Table 2:** Top variants with significant distribution difference in SCD cases than in healthy controls.

CHR	Gene	SNP	Variant Type	MAF (Cases)	MAF (Controls)	*p*-Value
1	*ACKR1*	rs12075 (A>G)	Missense	0.010	0.217	9.35 × 10^−13^
		rs12074934 (T>G)	Intergenic	0.487	0.218	1.71 × 10^−9^
		rs863002 (C>T)	Regulatory (CTCF site)	0.010	0.176	4.45 × 10^−11^
6	*AGER*	rs1800684 (T>A)	Upstream	0.017	0.122	2.44 × 10^−8^
1	*FCER1A*	rs2494250 (C>G)	Intron (500 B downstream)	0.011	0.174	3.31 × 10^−11^
11	*HBBP1*, *HBD*	rs2071348 (T>G)	Intron	0.467	0.164	6.77 × 10^−12^
11	*HBE1*, *HBG2*	rs2213170 (G>A)	Intron	0.440	0.015	1.78 × 10^−11^
		rs7130110 (C>G)	Regulatory	0.466	0.179	9.21 × 10^−11^
		rs2213169 (G>A)	Intron	0.437	0.015	2.17 × 10^−11^
11	*HBG2*	rs2236794 (C>T)	Downstream	0.070	0.639	4.68 × 10^−46^
6	*HLA-A*	rs2844806 (T>C)	Intergenic	0.330	0.536	5.92 × 10^−9^
	*HLA-G*	rs2524035 (G>A)	Intron	0.095	0.239	2.85 × 10^−8^
	*HLA-DRB1/HLA-DQB1*	rs3135006 (C>T)	Intergenic	0.082	0.294	8.82 × 10^−14^
		rs2395522 (T>A)	Intergenic	0.409	0.617	8.00 × 10^−9^
11	*MMP26*	rs6578521 (G>A)	Intron	0.069	0.510	1.38 × 10^−33^
		rs11822851(A>G)	Intron	0.466	0.141	2.06 × 10^−13^
1	*MPTX1/CADM3-AS1*	rs3845624 (C>A)	Intergenic	0.018	0.233	8.35 × 10^−15^
6	*NOTCH4*	rs3132946 (G>A)	Intron	0.016	0.143	1.02 × 10^−9^
		rs3132940 (G>T)	Intron	0.016	0.140	1.45 × 10^−9^
		rs3096702 (G>A)	Upstream	0.077	0.222	7.92 × 10^−9^
		rs9267898 (C>T)	Intergenic	0.063	0.209	1.65 × 10^−9^
1	*OR10J8P/OR10J9P*	rs6687840 (T>C)	Intergenic	0.182	0.453	2.31 × 10^−15^
		rs4446959 (C>T)	Intergenic	0.180	0.459	3.17 × 10^−16^
11	*OR51B5*	rs147062602 (CAGCCCCAG9GTCTGTGG>ins)	Frameshift	0.351	0.018	5.75 × 10^−10^
		rs10838058 (A>G)	Intron	0.097	0.374	1.66 x10^−18^
		rs10837853 (G>A)	Intron	0.306	0.611	3.82 × 10^−16^
		rs78253695 (GTC>del)	Intron	0.114	0.342	6.78 × 10^−14^
		rs180750244 (G>A)	Intron	0.017	0.153	3.02 × 10^−10^
11	*OR51S1*	rs12361955 (A>G)	Missense	0.077	0.425	2.09 × 10^−25^
11	*OR51V1*	rs7933549 (G>A)	Missense	0.484	0.015	1.95 × 10^−12^
11	*OR52A1*	rs112098990 (C>del)	Frameshift	0.029	0.308	9.87 × 10^−20^
11	*OR52A5*	rs2472530 (A>G)	Missense	0.453	0.150	6.73 × 10^−12^
5	*POC5*	rs2307111 (C>T)	Missense	0.397	0.592	4.47 × 10^−8^
11	*RRM1*	rs55945048 (T>C)	Downstream	0.355	0.101	1.61 × 10^−9^
6	*SCAND3*	rs450630 (A>G)	Missense	0.357	0.552	4.71 × 10^−8^
11	*SIDT2*	rs10535646 (TGC>del)	Upstream	0.296	0.497	9.81 × 10^−9^
11	*STIM1*	rs10767695(A>G)	Intron-NMD	0.597	0.300	4.23 × 10^−11^
		rs7120828 (C>T)	Intron-NMD	0.401	0.161	2.47 × 10^−8^
		rs4243966 (T>C)	Intron-NMD	0.044	0.223	1.04 × 10^−12^
11	*TRIM5*	rs10838525 (C>T)	Missense	0.050	0.267	2.26 × 10^−15^
		rs11038628 (C>T)	Missense	0.330	0.074	7.03 × 10^−10^
		rs12786650 (T>C)	Intron	0.078	0.337	8.82 × 10^−18^
		rs57956987 (T>C)	Intron	0.030	0.165	4.84 × 10^−10^
11	*TRIM6*	rs3740999 (A>C)	Splice donor	0.075	0.283	1.45 × 10^−13^
		rs11038294 (C>T)	Intron	0.107	0.282	4.55 × 10^−10^
		rs12272467 (A>G)	Regulatory (TF site)	0.600	0.255	1.76 × 10^−14^
11	*TRIM22*	rs67573252 (T>G)	Intron-NMD	0.050	0.228	2.54 × 10^−12^
11	*TRIM 34*	rs2342380 (A>G)	Intron	0.136	0.345	1 × 10^−11^

CHR: chromosome; SNP: single nucleotide polymorphism; MAF: minor allele frequency.

## Data Availability

The original data of this study are included in the article. However, some detailed genotyping data are unavailable due to privacy aspects. Further inquiries can be directed to the corresponding author.

## Supplementary Materials

The following supporting information can be downloaded at: https://www.mdpi.com/article/10.3390/ijms26062817/s1. References [31,32,33,37,44,48,49,53,54,55,56,57,58,68,69,77,78,79,80,81,82,83,84,85,86,87] are cited in Supplementary Materials.
