# Supplementary material for: Unraveling the Complex Genomic Interplay of Sickle Cell Disease Among the Saudi Population: A Case-Control GWAS Analysis
Source: Int J Mol Sci. 2025 Mar 20;26(6):2817. doi: 10.3390/ijms26062817 (PMC11942740; doi:10.3390/ijms26062817)
Supplement: Supplementary file 1 [file ijms-26-02817-s001.zip › ijms-3468091-supplementary.pdf]

**Supplementary Table S1: Overview of Known Genes Characterizing the Individuals with Sickle Cell Disease**

| Gene (Protein) | Potential Pathways Linked with Hemoglobinopathies | Reported Phenotypes |
|---|---|---|
| **Olfactory receptor (OR) gene clusters:** OR51V1, OR52A1, OR52A5, OR51B5, OR51S1, OR10J8P, and OR10J9P. | Expressed in erythroid cells near the β-globin cluster, may play a regulatory role in hemoglobinopathies [31–33]. | **Hematological Measurements:**<br>Hemoglobin (Hgb) measurement, Hematocrit, Erythrocyte count,<br>Mean corpuscular volume (MCV),<br>Red blood cell distribution width (RDW),<br>Mean corpuscular hemoglobin concentration (MCHC),<br>Mean reticulocyte volume (MRV),<br>Fetal hemoglobin (HbF) measurement,<br>Hgb A1 & A2 measurement.<br><br>**Leukocyte and Platelet Measurements:**<br>Neutrophil count, Leukocyte count,<br>Mean platelet volume (MPV)<br><br>**Hemolysis and Hematological Conditions:**<br>Hemolysis, Thromboembolism in SCD,<br>Venous thromboembolism, Ischemia<br>Ischemic cardiomyopathy.<br><br>**Inflammatory Markers:**<br>C-reactive protein (CRP) levels<br>Monocyte chemoattractant protein-1 levels<br><br>**Additional Conditions:**<br>Various SCD severity levels |
| **TRIM Family** (Tripartite motif-containing protein): TRIM5, TRIM6, TRIM22, and TRIM34. | Involved in innate immune modulation, cell cycle progression, and transcriptional regulation. This suggests an effect on inflammation, oxidative stress, hematopoietic stem cell differentiation, and immune responses, all of which are critical for SCD and its complications [37].<br>TRIM family proteins activate transforming growth factor beta (TGF-β)-activated kinase 1 (TAK1) to induce NF-κB and MAP kinase signaling, also promote NF-κB activation through IKKβ ubiquitination, and regulate AP-1 signaling, antiviral responses, and innate immune activities [37]. | **Hematological Measurements:**<br>MPV, RBC density, MCHC, Erythrocyte count, Platelet count, MCH, RDW, Hgb measurements.<br><br>**Inflammatory and Cardiovascular Conditions:**<br>Various inflammatory conditions<br>Cardiovascular disease (CVD)<br>Coronary artery disease (CAD)<br>Thromboembolism in SCD<br>Intracerebral hemorrhage<br><br>**Lipid and Liver Function:**<br>Lipid Profiles<br>Bilirubin measurements<br>Liver function<br><br>**Additional Conditions:**<br>Anemia, Kidney dysfunction |

| | | |
|---|---|---|
| ***ACKR1***<br>(Atypical Chemokine Receptor 1) | Known also as DARC, involved in inflammation and chemokine regulation. It may affect the severity and organ damage, including leg ulcers and renal function [55,56]. | **Inflammatory Markers:**<br>MCP-1 (Monocyte Chemoattractant Protein-1), CCL2, Interleukin-8 (IL-8), Chemokines,<br>White blood cell (WBC) measurement<br><br>**Additional Conditions:**<br>Anemia, Leg Ulcers, Priapism, Kidney dysfunction |
| **AGER**<br>(The advanced glycosylation end product receptor) | Critical mediator of pro-inflammatory signaling in SCD, contributing to vascular dysfunction and the pathogenesis of vaso-occlusive crisis (VOC) [77]. | **Hematological Measurements:**<br>MPV, Erythrocyte counts,<br>Reticulocyte measurements<br><br>**Inflammation:**<br>Inflammatory Biomarkers<br>Adhesion Molecules<br><br>**Additional Conditions:**<br>Acute chest syndrome (ACS), pneumonia, or respiratory infection, Acute pain episode, Cerebrovascular disease |
| **HLA-gene family**<br>(Human leukocyte antigen): A, DQB1, DRB1, and G. | Encodes the major histocompatibility complex proteins displaying peptides to immune cells for self/non-self-discrimination. Certain HLA variants are associated with particular SCD complications involving VOC and organ damage through driving aberrant immune cell activation [78–81]. | **Hematological Measurements:**<br>Hgb Measurement, MPV<br>Mean reticulocyte measurement<br><br>**Inflammatory Markers:**<br>Leukocyte cell adhesion<br>Regulation of leukocyte differentiation<br>Inflammatory response to antigenic stimulus<br><br>**Additional Conditions:**<br>ACS, pneumonia, or respiratory infection<br>Acute pain episode, HbF level<br>Hyperbilirubinemia, Cholelithiasis, Cholecystitis, or Cholecystectomy |
| **FCER1A**<br>(Fc Epsilon Receptor Ia) | Encodes the alpha chain of the high-affinity IgE receptor (FcεRI), primarily expressed on mast cells and basophil surfaces. This receptor plays a crucial role in allergic responses and the immune system's reaction to parasitic infections [82]. | **Inflammatory Markers:**<br>CRP level,<br>C-C motif chemokine ligand 2 (CCL2) measurement, Serum IgE measurement,<br>Chemokine measurement,<br>Adhesion molecules |

| | | |
|---|---|---|
| **MMP26**<br>(Matrix metallopeptidase 26) | Also called matrilysin-2, an enzyme responsible for the degradation of extracellular matrix components (ECM), an important process in angiogenesis, inflammation, and tissue remodeling [44]. In the context of SCD, MMP26 may indirectly mediate various pathogenic processes contributing to the condition's complications. | **Hematological Measurements:**<br>Hgb Level, MCV, MCH, RDW,<br>Erythrocyte Counts,<br>Thromboembolism in SCD<br><br>**Biomarker:**<br>Cystatin C levels (kidney function marker)<br><br>**Other conditions:**<br>Pneumonia,<br>Cognitive performance assessments<br>Ischemic stroke (time-to-first event) |
| **HBB (β-globin) locus**<br>(HBBP1, HBD, HBE1, HBG2) | Located on chromosome 11, each gene contributes to hemoglobin production and regulation. HBBP1 is thought to have regulatory roles, HBD is responsible for producing delta-globin in adults, HBE1 is essential in early embryonic development, and HBG2 is vital for fetal hemoglobin. Alterations in these genes can impact hemoglobinopathies, reflecting the complex interactions within the HBB locus [83]. | **Hematological Measurements:**<br>Reticulocyte count<br>MCV<br>MCHC<br>HbF levels<br>Thromboembolic events SCD<br>Hemolysis |
| **NOTCH4**<br>(Notch Receptor 4) | A member of the NOTCH family that regulates endothelial function, inflammation, and hematopoiesis. Dysfunctional NOTCH4 significantly impacts vascular integrity and immune responses by inducing inflammatory biomarkers and influencing endothelial function. Hence, it may be potentially linked with different SCD phenotypes [68,69]. | **Hematological Measurements:**<br>Hgb measurement, Platelet count,<br>Reticulocyte count, Hematocrit<br><br>**Inflammatory Conditions:**<br>Inflammatory biomarkers and leukocyte measurement<br><br>**Other Conditions:**<br>Nephropathy, Asthma, Cerebrovascular disease (stroke) |
| **POC5**<br>(POC5 Centriolar Protein) | A protein at the centrosome, a key component in cell division and the organization of microtubules in the cell [84].<br>There is no established particular role of POC5 with hemoglobinopathy pathways. | **No related hematological traits reported**<br>WBC count<br>Metabolic biomarkers<br>Blood pressure measurement |
| **RRM1**<br>(Ribonucleotide Reductase Catalytic Subunit M1) | Encodes the ribonucleotide reductase enzyme, which plays an important role in DNA synthesis through the conversion of ribonucleotides to deoxyribonucleotides. There are significant connections between RRM1 and hydroxyurea resistance. Specifically, overexpression of both RRM1 and RRM2, which are targeted by hydroxyurea [48,49]. | **Hematological Measurements:**<br>MCV, RDW, MCH<br><br>**Immune Parameters:**<br>Lymphocyte count |
| **SIDT2**<br>(SID1 Transmembrane Family Member 2) | A protein-coding gene that plays a significant role in regulating lipid metabolism and immune response [53]. It is also crucial for maintaining kidney structure and function, as demonstrated in knockout animal models [85]. Direct evidence linking this protein to SCD is still lacking, but it may influence various SCD outcomes, such as hepatic disorders, cardiovascular dysfunctions, or SCD nephropathy, through its roles in immune regulation, lipid metabolism, and maintaining kidney structure and function. | **Lipid Measurements:**<br>High-Density Lipoprotein (HDL) Cholesterol Levels<br>Triglycerides Measurements<br><br>**Hematological Measurements:**<br>Platelet Count<br>CVD<br>CAD |

| | | |
|---|---|---|
| **STIM1**<br>(Stromal Interaction Molecule 1) | Essential in maintaining calcium ion (Ca++) entry into cells through calcium-release activated calcium (CRAC) channels when intracellular calcium levels are low [86]. No direct link to SCD in the literature, but STIM1's role in the Ca++ signaling pathway, inflammation, and cellular functions could suggest an influence on SCD outcomes [54]. | **Hematological Measurements:**<br>Hgb Measurement<br>PMPV<br>MCV<br>MRV<br>Hematocrit<br><br>**Other condition:**<br>Lung Function (FEV1/FVC) |
| **SCAND3**<br>(SCAN Domain Containing 3) | Coding for a protein with nucleic acid binding activity, has a significant role in cell cycle regulation and epithelial cell proliferation. Recent findings indicate a role for SCAND3 in blood cell formation and function [57,58]. | **Hematological Measurements:**<br>Hgb Measurement<br>Platelet Count<br>Metabolic Biomarker |
| **MPTX1, CADM3-AS1**<br>(Cell Adhesion Molecule 3) | CADM3 is a protein involved in cell adhesion and signaling within the vascular system. Its role in SCD may potentially influence disease manifestations by disrupting these processes, leading to abnormal blood flow and increased inflammation [87]. | **Inflammatory biomarkers**<br>CCL2 levels<br>CRP levels<br>Other inflammatory biomarkers |

Reported Phenotypes from the GWAS Catalog & Previously Published Comprehensive Systematic Review & Meta-Analysis (2023) [12].
